# Revelation of Gordon Syndrome: A Case of Persistent Hyperkalemia

**DOI:** 10.7759/cureus.84341

**Published:** 2025-05-18

**Authors:** Bosky Modi, Prince Modi, Freny Patel, Godfrey Tabowei, Anand Reddy

**Affiliations:** 1 Internal Medicine, Texas Tech University Health Sciences Center, Odessa, USA; 2 Internal Medicine, Pramukhswami Medical College, Anand, IND; 3 Nephrology, Permian Basin Kidney Center, Odessa, USA

**Keywords:** genetic analysis, gordon syndrome, persistent hyperkalemia, pseudohypoaldosteronism, wnk1 gene

## Abstract

We present the case of a 14-year-old male with a novel diagnosis of Gordon’s syndrome (GS, pseudohypoaldosteronism type 2), notable for its atypical presentation in the absence of hypertension. The patient exhibited persistent hyperkalemia, metabolic acidosis, and normotension, prompting genetic testing that identified a heterozygous mutation in the *WNK1* gene. Subsequent evaluation of the patient’s father, who had no history of hypertension but demonstrated similar biochemical abnormalities, revealed the same genetic variant, confirming the diagnosis in both individuals. The lack of hypertension in both cases deviates from the classic phenotype of GS. Treatment with low-dose thiazide diuretics led to gradual correction of the electrolyte disturbances, supporting the diagnosis and highlighting phenotypic variability within this rare condition.

## Introduction

Gordon syndrome (GS), also known as pseudohypoaldosteronism type 2 or familial hyperkalemia and hypertension syndrome, is a rare inherited disorder characterized by low-renin hypertension, hyperkalemia, and metabolic acidosis, particularly type IV renal tubular acidosis [1]. The condition arises due to excessive activation of the thiazide-sensitive Na/Cl cotransporter (NCC) in the distal convoluted tubules, leading to increased NaCl reabsorption, reduced excretion of K⁺ and H⁺, and subsequent manifestations including high blood pressure, hyperkalemia, hyperchloremic metabolic acidosis, suppressed renin levels, and variable aldosterone levels. Clinical symptoms of GS encompass a spectrum including growth difficulties, myasthenia, periodic paralysis, skeletal abnormalities, and psychomotor slowness [2].

The age of diagnosis for GS varies widely, ranging from 7 months to 39 years in certain studied cohorts. There is no established correlation between the severity of biochemical abnormalities and age or blood pressure elevation. While most families exhibit an autosomal dominant inheritance pattern, recent research indicates that some traits of GS can be inherited in an autosomal recessive manner. The condition stems from four recognized monogenic causes: *WNK1*, *WNK4*, *KLHL3*, and *CUL3* [1]. These genes play pivotal roles in regulating renal ion transporters, and mutations in any of them can disrupt normal ion transport mechanisms, contributing to the pathophysiology of GS.

## Case presentation

A 14-year-old previously healthy male presented to the ED with a five-day history of epigastric abdominal pain, accompanied by frequent episodes of non-bloody vomiting and watery diarrhea occurring three to four times per day. He denied recent travel or contact with sick individuals. On examination, his systolic blood pressure was below the 95th percentile for age, consistent with low-normal blood pressure in pediatric patients. His heart rate was 92 beats per minute, and he was afebrile, with a normal respiratory rate and oxygen saturation. Physical examination revealed dry oral mucosa, suggesting mild dehydration, but was otherwise unremarkable.

The initial laboratory assessment showed a normal complete blood count with WBC count 7.9 x10³/microL (normal range, 4.5-11 x10³/microL), normal differential, hemoglobin 13.0 g/dL (normal range, 13-17 g/dL), and platelets 232 x10³/microL (normal range, 150-450 x10³/microL). Renal function tests were normal, with serum blood urea nitrogen (BUN) 9 mg/dL (normal range, 6-20 mg/dL) and creatinine 0.9 mg/dL (normal range, 0.7-1.2 mg/dL). Liver function tests were normal, with aspartate aminotransferase (AST) 23 U/L (normal range, 10-40 U/L), alanine aminotransferase (ALT) 30 U/L (normal range, 10-40 U/L), alkaline phosphatase (ALP) 78 U/L (normal range, 30-300 U/L), albumin 3.6 g/dL (normal range, 3.5-5 g/dL), total protein 6.5 g/dL (normal range, 6-8.3 g/dL), and total bilirubin 0.3 mg/dL (normal range, 0.1-1.2 mg/dL). Electrolytes showed normal sodium at 141 mEq/L (normal range, 135-145 mEq/L), normal calcium at 8.9 mg/dL (normal range, 8.6-10 mg/dL), mild hyperchloremia at 109 mEq/L (normal range, 98-108 mEq/L), mild hyperkalemia at 5.6 mEq/L (normal range, 3.5-5.2 mEq/L), and metabolic acidosis with bicarbonate 19 mg/dL (normal range, 22-29 mg/dL) and a normal anion gap of 13 mEq/L (normal range, 10-14 mEq/L). The blood sample was confirmed to be a non-hemolyzed specimen. Serum AM cortisol was 10.8 mg/dL (normal range, 5-25 mg/dL). Stool gastrointestinal polymerase chain reaction (PCR) testing was negative for bacterial pathogens. A CT scan of the abdomen and pelvis and an electrocardiogram were unremarkable. His symptoms were suspected to be due to viral gastroenteritis, and he was symptomatically treated with bowel rest, IV fluids, and antiemetics. His symptoms subsided after two days, but repeat laboratory blood work showed a potassium level of 6.1 mEq/L, bicarbonate of 19 mg/dL, and chloride of 108 mEq/L. Plasma renin was 1.2 ng/mL/hour (normal range, 0.2-1.6 ng/mL/hour), aldosterone was 4.6 ng/dL (normal <15 ng/dL), and 24-hour urinary potassium excretion was 52 mEq/day (normal range, 25-125 mEq/day). Table 1 summarizes relevant laboratory values at initial presentation and 48 hours later. An electrocardiogram remained unremarkable.

**Table 1:** Laboratory values at initial presentation and 48 hours later. SI units: Système International; Na⁺: Sodium; K⁺: Potassium; Cl⁻: Chloride; Ca²⁺: Calcium; CO₂: Bicarbonate; mEq: Milliequivalent; mmol: Millimole; mg: Milligram; L: Liter; dL: Deciliter; mL: Milliliter; ng: Nanogram.

Type of Sample	Parameter	Initial Value	Subsequent Value	Normal Range (SI and Conventional Units)
Blood	Na⁺ (Sodium)	141	143	135-145 mEq/L or mmol/L
	K⁺ (Potassium)	5.6	6.1	3.5-5.2 mEq/L or mmol/L
	Cl⁻ (Chloride)	109	108	98-108 mEq/L or mmol/L
	Ca²⁺ (Calcium)	8.9	9.5	8.6-10 mg/dl (2.15-2.50 mmol/L)
	CO₂ (Bicarbonate)	19	19	22-29 mg/dl (22-29 mmol/L)
	Anion Gap	13	-	10-14 mEq/L or mmol/L
	Creatinine	0.9	0.8	0.7-1.2 mg/dl (2.1-7.1 mmol/L)
	Urea (BUN)	9	6	6-20 mg/dl (0.33-1.11 mmol/L)
	Renin	1.2	-	0.2-1.6 ng/mL/hour
	Aldosterone	4.6	-	<15 ng/dL
	Cortisol (AM)	10.8	-	5-25 mcg/dl (138-690 nmol/L)
24-hour Urine	Volume	-	2000	800-2000 mL/day
	Osmolality	-	425	500-850 mOsm/kg
	Potassium (24h excretion)	-	52	25-125 mEq/day or mmol/day
	Potassium Concentration	-	26	20-40 mEq/L or mmol/L

On further history taking, his father reported having years of asymptomatic persistent hyperkalemia and normal anion gap metabolic acidosis with normotension. Genetic analysis was performed during outpatient follow-up and identified a heterozygous pathogenic missense mutation (c.897G>A, His160Arg) in the WNK1 gene in both father and son, diagnostic of GS. Before discharge, the patient was treated with an oral potassium binder to maintain potassium levels below 6 mEq/L until the genetic test results were available.

The absence of hypertension in both individuals contrasted with the typical presentation of GS. They were treated with hydrochlorothiazide (HCTZ) 12.5 mg daily, resulting in gradual normalization of hyperkalemia and metabolic acidosis after six weeks of treatment in both the patient and his father. At the 3-month follow-up, potassium and bicarbonate levels remained within normal ranges, and both the patient and his father denied any hypotensive events or adverse effects from HCTZ.

## Discussion

GS, also known as pseudohypoaldosteronism type II, is a rare inherited disorder characterized by hypertension, hyperkalemia, metabolic acidosis, and normal renal function [1]. It is caused by mutations in genes that regulate sodium-chloride cotransport in the distal nephron, including *WNK1*, *WNK4*, *KLHL3*, and *CUL3* [1]. These mutations lead to increased sodium reabsorption and impaired potassium and hydrogen ion excretion, explaining the characteristic clinical features [2].

The identified mutation in the *WNK1* gene, specifically the c.897G > A alteration resulting in the substitution of the 160th histidine residue with arginine (His160Arg), contributes to the clinical manifestations observed. The mutated gene locus segregates GS into four subtypes, delineated as: (a) 1q31-42, (b) 17p11-q21, (c) 12p13, and (d) unknown locus [2]. Mutations primarily influence the tissue distribution in the kidneys in the second and third subtypes, involving WNK4 and *WNK1*, which are expressed in the distal nephron [2].

Further exploration of the roles of *WNK1*, *WNK4*, and *CUL3* in regulating the thiazide-sensitive sodium-chloride symporter (NCC) highlights their distinct functions [3-5]. The Renal Outer Medullary Potassium channel (*ROMK*) and Epithelial Sodium Channel (ENaC) are key downstream effectors: WNK4 promotes paracellular chloride reabsorption but inhibits NCC activity, *ROMK* (potassium excretion), and ENaC (sodium reabsorption). In contrast, *WNK1* inhibits *ROMK* activity while enhancing NCC, ENaC, and paracellular chloride reabsorption, and it also inhibits WNK4. These changes reduce potassium excretion by increasing sodium reabsorption through NCC, leaving less sodium available for potassium exchange in the distal renal tubules [6-11]. This disrupts the lumen's negative charge, further reducing potassium excretion via *ROMK* channels [6, 8-12]. The reduced negative lumen charge also impairs hydrogen ion excretion, contributing to metabolic acidosis, particularly in the context of hyperkalemia [9, 13, 14].

Plasma and urine aldosterone levels typically remain within the normal range due to suppressed renin activity, although hyperkalemia independently tends to stimulate aldosterone secretion [6, 9-11]. The severity of clinical phenotypes varies among GS patients depending on the underlying gene mutation, with CUL3 mutations generally associated with more severe manifestations compared to *KLHL3*, *WNK4*, and *WNK1* mutations. Hyperkalemia is the most common clinical feature in GS patients, followed by hypertension and metabolic acidosis [4, 15]. However, this case highlights atypical features, most notably the absence of hypertension, indicating that GS should be considered even in young patients who lack its classic symptoms but present with persistent hyperkalemia [16].

Low-dose thiazide diuretics are effective in alleviating clinical symptoms in GS by inhibiting NCC activity in the distal nephron. Previous research has demonstrated their efficacy in correcting hypertension, hyperkalemia, and metabolic acidosis in affected individuals [4, 15]. In this case, both the father and son were treated with hydrochlorothiazide and tolerated the therapy without any hypotensive events. This case underscores the importance of considering GS in pediatric patients presenting with unexplained hyperkalemia, even in the absence of hypertension or metabolic acidosis. Comprehensive genetic testing and an understanding of the disorder’s complex pathophysiology are essential to guide effective treatment and long-term management.

## Conclusions

This case highlights an atypical presentation of GS in a 14-year-old boy and his father, both carrying a *WNK1* gene mutation yet lacking the hallmark symptom of hypertension. The presence of persistent hyperkalemia, hyperchloremic metabolic acidosis with a normal anion gap, and suppressed plasma renin activity prompted genetic investigation, ultimately confirming the diagnosis. Despite the absence of elevated blood pressure, the biochemical abnormalities were consistent with pseudohypoaldosteronism type 2, and the clinical response to low-dose thiazide therapy further supported the diagnosis.

These findings underscore the phenotypic variability of GS and emphasize the importance of including it in the differential diagnosis of unexplained hyperkalemia in normotensive patients. Early recognition and targeted therapy, even in the absence of hypertension, can result in favorable clinical outcomes and help prevent complications. This case reinforces the value of genetic testing and a comprehensive understanding of renal electrolyte handling in diagnosing and managing rare tubulopathies.
